# Blood stasis may cause thrombosis in the left superior pulmonary vein stump after left upper lobectomy

**DOI:** 10.1186/s13019-014-0159-8

**Published:** 2014-09-18

**Authors:** Kazuto Ohtaka, Yasuhiro Takahashi, Satoko Uemura, Yasuhito Shoji, Satoshi Hayama, Tatsunosuke Ichimura, Naoto Senmaru, Yasuhiro Hida, Kichizo Kaga, Yoshiro Matsui

**Affiliations:** Department of Thoracic Surgery, Steel Memorial Muroran Hospital, 1-45 Chiribetsu-cho, Muroran, 050-0076 Hokkaido Japan; Department of Cardiovascular and Thoracic Surgery, Hokkaido University Graduate School of Medicine, Sapporo, Hokkaido Japan

**Keywords:** Lobectomy, Thrombosis, Pulmonary vein stump, Ultrasonography, Spontaneous echo contrast

## Abstract

**Background:**

We previously reported that arterial infarction of vital organs after lobectomy might occur only after left upper lobectomy and be caused by thrombosis in the left superior pulmonary vein stump. We hypothesized that changes in blood flow, such as blood stasis and disturbed stagnant flow, in the left superior pulmonary vein stump cause thrombosis, and this was evaluated by intraoperative ultrasonography.

**Methods:**

From July 2013 to April 2014, 24 patients underwent lobectomy in the Steel Memorial Muroran Hospital. During the procedure, an ultrasound probe was placed at the pulmonary vein stump and the velocity in the stump was recorded with pulse Doppler mode. The peak velocity and the presence of spontaneous echo contrast in the stump were evaluated. After the operation, the patients underwent contrast-enhanced CT within 3 months.

**Results:**

The operative procedures were seven left upper lobectomies, four left lower lobectomies, seven right upper lobectomies, and six right lower lobectomies. Blood flow was significantly slower in the left superior pulmonary vein stump than in the right pulmonary vein stumps. However, that was not significantly slower than that in the left inferior pulmonary vein stump. Spontaneous echo contrast in the pulmonary vein stump was seen in three patients who underwent left upper lobectomy. Of the three patients with spontaneous echo contrast, two patients developed thrombosis in the left superior vein stump within 3 months after the operation. There was no patient who developed arterial infarction.

**Conclusions:**

In patients who underwent left upper lobectomy, intraoperative ultrasonography to evaluate blood flow and the presence of spontaneous echo contrast in the left superior pulmonary vein stump may be useful to predict thrombosis that may cause arterial infarction.

**Electronic supplementary material:**

The online version of this article (doi:10.1186/s13019-014-0159-8) contains supplementary material, which is available to authorized users.

## Background

Arterial infarction of vital organs, such as the brain, kidneys, and intestines, is a very important and serious complication after pulmonary lobectomy. There have been 12 reported cases of arterial infarction of vital organs after lobectomy [1]-[11]. All 12 cases underwent left upper lobectomy (LUL). Therefore, we hypothesized that LUL predispose patient to arterial infarction.

What is the cause of arterial infarction after LUL? Of the previously reported 12 cases, six cases showed a thrombus in the left superior pulmonary vein (LSPV) stump [1]-[6]. Therefore, thrombosis in the LSPV stump might cause arterial embolism after LUL. We previously investigated thrombosis in the pulmonary vein (PV) stump after lobectomy [12],[13]. A thrombus in the PV stump was detected in 3.3-3.6% of all patients and occurred only in patients who underwent LUL but never in patients with other lobectomies. Among the patients who underwent LUL, a thrombus was detected in 13.5-17.9%. Ichimura et al. also reported in their case report that a thrombus was detected in 3.4% of the patients who underwent LUL [5]. Thrombosis in the pulmonary vein stump occurs with a high frequency after LUL and may cause arterial embolism.

What is the cause of thrombosis in the LSPV stump? As for thrombosis in the pulmonary artery (PA) stump, there have been some reports that thrombosis developed more frequently after right pneumonectomy than after left pneumonectomy because the right PA stump was anatomically longer than the left PA stump [14]-[16]. We previously reported that the LSPV stump remained significantly longer than the other three PV stumps [12]. We hypothesized that changes in blood flow, such as blood stasis and disturbed stagnant flow, in the long PV stump caused thrombosis [13]. In this study, blood flow in the PV stump after lobectomy was evaluated using intraoperative ultrasonography to validate this hypothesis.

## Methods

This study was approved by the ethics committee of the Steel Memorial Muroran Hospital. Informed consent was obtained from all patients and documented in a writing form before the surgery.

### Patients

From July 2013 to April 2014, 36 patients underwent lobectomy in the Steel Memorial Muroran Hospital. The following patients were excluded: four patients who underwent right middle lobectomy (RML); two patients whose respiratory status exacerbated on one-lung ventilation; two patients who underwent long surgery for more than 6 hours; two patients with allergy to contrast dye; one patient with interstitial pneumonia; and one patient whose PV was divided in the pericardium. Finally, 24 patients were the subjects of this study. In these patients, blood flow in the PV stump during the operation was evaluated by the method described below, and they underwent contrast-enhanced chest computed tomography (CT) within 3 months after surgery.

### Operative procedure

For all patients, except those with large tumor size, lymph node metastases, chest wall invasion, bronchial invasion, or pulmonary vessel invasion on preoperative images, video-assisted thoracoscopic surgery (VATS) with a 7-cm, small thoracotomy, 3-cm window, and two ports was performed. For the other patients, open chest surgery with a 15 to 20-cm thoracotomy was performed.

Under one-lung ventilation in the lateral position, the operation was performed as follows. First, the PV was divided using a linear surgical stapler. Second, a few branches of the PA were divided by ligation or linear staplers. Third, the bronchus was divided using a linear stapler. Fourth, dissection of mediastinal lymph nodes was performed. Then, blood flow in the PV stump was evaluated by ultrasonography using the method described below. Finally, a chest drainage tube was inserted, and the operation was completed after closure of all wounds.

### Perioperative management

In patients receiving warfarin, the warfarin was discontinued from 7 days before surgery, and then they were started on intravenous heparin for up to 6 hours before surgery. On postoperative day (POD) 1, heparin was re-started if there was no sign of bleeding. On POD 7, oral warfarin was re-started after removal of the epidural catheter. If the prothrombin time entered the therapeutic range, heparin was stopped.

In the patients receiving antiplatelet drugs, cardiovascular or neurosurgical specialists were consulted to determine whether that drug could be discontinued. If antiplatelet drugs could be discontinued, they were discontinued from about 7 days before surgery. In high-risk patients, heparin was administered preoperatively based on the advice of a specialist. Perioperative management of heparin was as previously described. Oral antiplatelet drugs were re-started after removal of the epidural catheter, and then heparin was discontinued.

### Evaluation of blood flow in the PV stump

**Figure 1:**
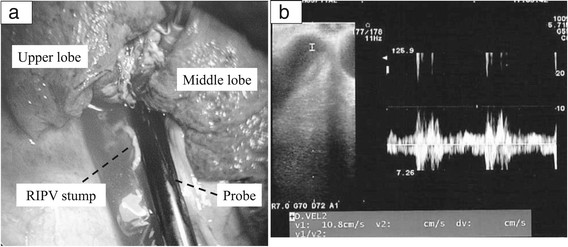
**Intraoperative ultrasonography.** An ultrasound probe is placed at the pulmonary vein stump **(a)**. The probe is carefully placed parallel to the staple line of the pulmonary vein stump. The velocity is recorded with pulse Doppler mode **(b)**. The peak velocity in the pulmonary vein stump is measured. RIPV: Right inferior pulmonary vein.

Blood flow in the PV stump was evaluated using an ultrasound console (ProSound Alpha 7, Hitachi Aloka Medical, Ltd., Tokyo, Japan) with a 33-mm, Large Animal Flexible Transducer (UST-5550, Hitachi Aloka Medical, Ltd.). A small amount of saline was infused into the thoracic cavity. An ultrasound probe was placed at the PV stump under thoracoscopic guidance (Figure 1). The probe was carefully placed parallel to the staple line of the PV stump. The affected lung was then expanded.

First, the velocity in the PV stump was recorded with pulse Doppler mode (Figure 1). The sample volume was set at a point 1 cm from the top of the PV stump. The peak velocity in the PV stump was measured. To minimize intraobserver variability, the velocity was measured three times in each patient, and the average value was calculated. Second, the presence of spontaneous echo contrast (SEC) in the PV stump was checked by two thoracic surgeons.

### Statistical analysis

Analysis of the blood flow was performed using the Mann Whitney U test. All statistical analyses were performed using Stat View 5.0 software (SAS Institute Inc., Cary, NC, USA). A p value of less than 0.05 was regarded as significant.

## Results

The characteristics of the 24 patients are shown in Table 1. The operative procedures were: LUL in seven, left lower lobectomy (LLL) in four, right upper lobectomy (RUL) in seven, and right lower lobectomy (RLL) in six patients, respectively.

**Table 1:** **Patients' characteristics**

	Left upper lobectomy (n = 7)	Left lower lobectomy (n = 4)	Right upper lobectomy (n = 7)	Right lower lobectomy (n = 6)
Age, years, median value (range)	69 (58-77)	78.5 (58-80)	66 (54-82)	74 (64-78)
Sex, n				
Male/Female	3/4	4/0	6/1	6/0
BMI, kg/m^2^, median value (range)	25.5 (19.2-27.5)	23.6 (22.4-34.1)	22.9 (17.9-32.1)	21.6 (19.2-26.2)
Brinkmann index, median value (range)	225 (0-980)	340.5 (0-870)	600 (0-2820)	660 (0-2500)
Co-existing disease, n (%)				
Ischemic heart disease	0 (0)	2 (50)	0 (0)	1 (17)
Cerebral infarction	1 (14)	1 (25)	1 (14)	1 (17)
Malignant tumor	0 (0)	0 (0)	3 (43)	3 (50)
Hypertension	4 (57)	3 (75)	3 (43)	4 (67)
Diabetes mellitus	2 (29)	0 (0)	1 (14)	2 (33)
Atrial fibrillation	1 (14)	2 (50)	0 (0)	0 (0)
Medication, n (%)				
Antithrombotic drug	2 (29)	3 (75)	2 (29)	4 (67)
Steroids	0 (0)	0 (0)	1 (14)	1 (17)
CEA, ng/mL, median value (range)	3.3 (1.1-21.6)	3.1 (1.8-8.3)	4.3 (2.1-14.5)	4.6 (3.7-22.5)
Diagnosis, n				
Primary cancer/Metastatic tumor/Benign	6/0/1	4/0/0	5/1/1	6/0/0
Tumor size, cm, median value (range)	25 (12-37)	29 (18-60)	26 (15-30)	29 (11-60)
Operative approach, n				
VATS/Open thoracotomy	5/1	3/1	7/0	5/1
Operative time, minutes, median value (range)	216 (146-321)	252 (200-260)	229 (208-280)	221 (185-277)
Bleeding, mL, median value (range)	60 (20-200)	85 (0-150)	70 (0-145)	108 (40-220)
Postoperative day to remove chest tube, median value (range)	3 (2-12)	3 (2-7)	4 (2-11)	4.5 (2-6)
Postoperative complications, n (%)				
Pulmonary fistula	1 (14)	0 (0)	2 (29)	1 (17)
Empyema	0 (0)	0 (0)	1 (14)	1 (17)
Atrial fibrillation	0 (0)	0 (0)	0 (0)	0 (0)

CEA: Carcinoembryonic antigen, VATS: video-assisted thoracoscopic surgery.

**Figure 2:**
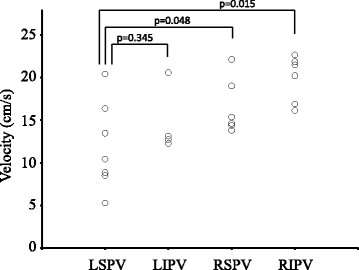
**Velocity in the pulmonary vein stump.** Blood flow is significantly slower in the left superior pulmonary vein stump than in the right pulmonary vein stumps. However, that is not significantly slower than in the left inferior pulmonary vein. LIPV: Left inferior pulmonary vein, LSPV: Left superior pulmonary vein, RIPV: Right inferior pulmonary vein, RSPV: Right superior pulmonary vein.

The blood flow was significantly slower in the LSPV stump (median, 10.4 cm/s; range, 5.3 to 20.4 cm/s) than in the right superior PV stump (median, 15.4 cm/s; range, 13.8 to 29.6 cm/s; p = 0.048) and the right inferior PV stump (median, 20.9 cm/s; range, 16.1 to 22.7 cm/s; p = 0.015; Figure 2). However, that was not significantly slower than in the left inferior PV stump (median, 12.9 cm/s; range, 12.3 to 20.6 cm/s; p = 0.345).

**Figure 3:**
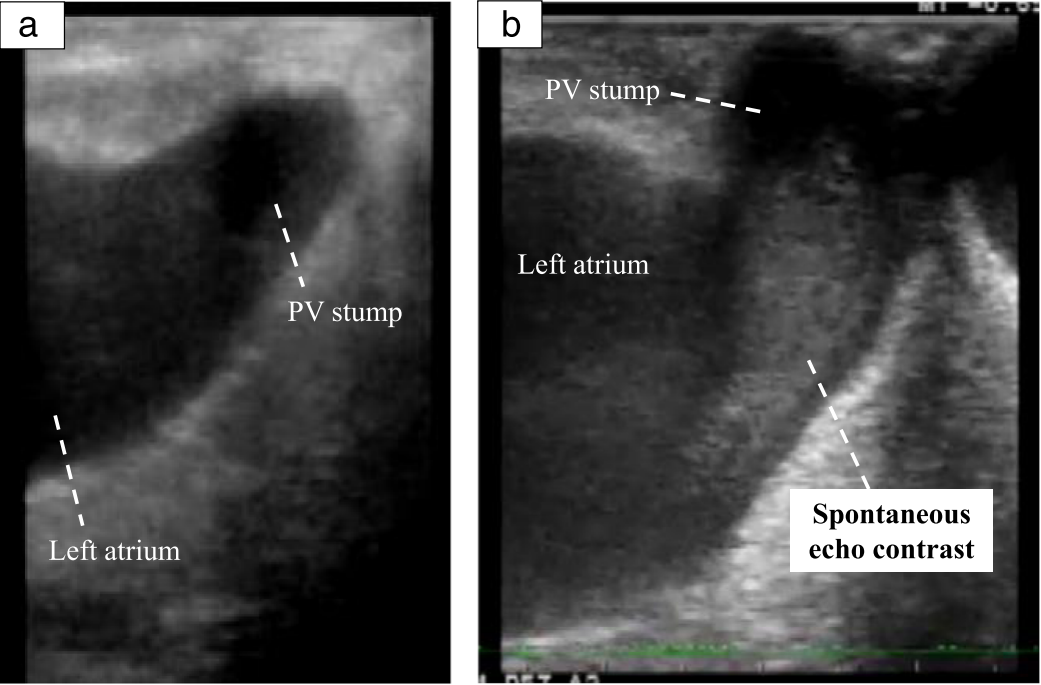
**Spontaneous echo contrast in the pulmonary vein stump.** Ultrasonographic images in the left superior pulmonary vein stump show the absence **(a)** and the presence of spontaneous echo contrast **(b)**. PV: Pulmonary vein.

**Figure 4:**
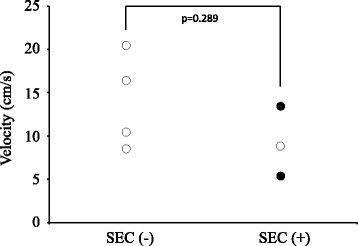
**A comparison of velocity of PV stump between SEC positive group and negative group in the patients who underwent LUL.** There was no significant difference of flow in the LSPV stump between SEC positive group and SEC negative group. Of the three patients with SEC, two patients developed thrombosis in the LSPV stump (black circle).

SEC was seen in the PV stump in three patients who underwent LUL (Figure 3). In the patients who underwent LLL, RUL, and RLL, there was no patients who were seen SEC. In the patients who underwent LUL, there was no significant difference of flow in the LSPV stump between SEC positive group and SEC negative group (p = 0.289; Figure 4).

**Figure 5:**
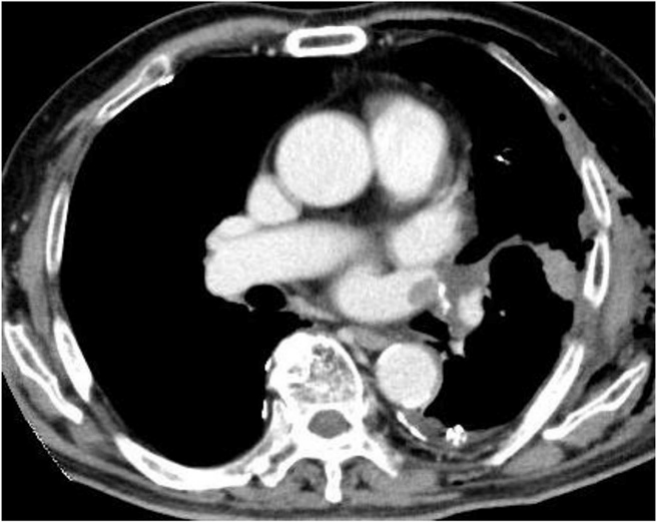
**Thrombosis in the left superior pulmonary vein stump.** In the patient who showed SEC in the left superior pulmonary vein stump on intraoperative ultrasonography, thrombosis in the stump is seen on contrast-enhanced computed tomography.

Of the three patients with SEC, two patients developed thrombosis in the LSPV stump within 3 months after the operation (Figures 4 and 5). Then, these patients were started anticoagulant therapy. In one patient, subsequent contrast-enhanced chest CT showed that the thrombus had disappeared. There was no embolic event in two patients.

## Discussion

We hypothesized that changes in blood flow, such as blood stasis and disturbed stagnant flow, in the long stump caused thrombosis in the LSPV stump after LUL [13]. That is, in a short PV stump, blood flow may occur because blood flow in the left atrium (LA) spreads through the entire PV stump. In a long PV stump, disturbed stagnant flow or blood stasis may occur because blood flow in the LA does not spread throughout the PV stump. In the right superior PV, because the branches to the upper lobe or middle lobe remain after RUL or RML, blood flow in the remaining branches spreads throughout the stump, and disturbed stagnant flow or blood stasis may not occur. In this study, blood flow was significantly slower in the LSPV stump than in the right PV stumps. SEC was detected in three patients who underwent LUL, and two of these patients developed thrombosis in the PV stump. This appears to support our hypothesis. However, in the patients who underwent LUL, there was no significant difference of flow in the LSPV stump between SEC positive group and SEC negative group. That may be caused by small number of sample. It is necessary to evaluate in large number of patients.

Of the 12 cases that had been reported with infarction of vital organs after lobectomy, one patient was found to have SEC in the LSPV stump on echocardiography [7]. Patients with atrial fibrillation (Af) or mitral valve stenosis have a tendency to develop a thrombus in the left atrial appendage (LAA) and cerebral infarction. In most of these patients, echocardiography shows SEC or smoke-like echoes in the LAA [17]. These echo findings are defined as follows: 1) it is composed of numerous microechoes; 2) it curls up slowly in the enlarged left atrial cavity; and 3) it vanishes as soon as it pours into the ventricular cavity [18]. These echo findings might reflect blood stasis in the LAA and demonstrate agglutination of red blood cells. We hypothesized that the same blood stasis occurred in the LSPV stump after LUL and predicted that SEC was detected in the LSPV stump by intraoperative ultrasonography. The result of the present study, that SEC was detected in the LSPV stump and the patients with SEC developed thrombosis, suggested that blood flow in the LSPV stump might become disturbed stagnant flow or develop stasis and cause thrombosis in the LSPV stump after LUL.

When does thrombosis in the LSPV stump develop after LUL? In paroxysmal Af, if it continues over 48 hours after onset, there is a need for anticoagulant therapy for defibrillation [19]. This means that a thrombus is more likely to be produced in LAA over 48 hours after the onset of Af. If one hypothesizes that a similar change in blood flow to Af occurs in the LSPV stump after LUL, a thrombus has the potential to develop within a few days after LUL. In fact, of 12 cases that had been reported, five cases developed infarction within 4 days after surgery. In the present study, two patients with thrombosis in the LSPV stump were diagnosed within postoperative 1 month. Therefore, it appears that thrombosis in the LSPV stump may develop within a few days after LUL.

How is thrombosis in the LSPV stump diagnosed? Generally, the D-dimer test is considered to be useful for diagnosing a thrombus. However, in our previous report, we demonstrated that the D-dimer level was normal in patients with thrombosis in the LSPV stump [12]. Thus, blood examination is considered not useful for early diagnosis. Transthoracic echocardiography may have difficulty carefully observing the PV stump after lung resection. Transesophageal echocardiography may be a painful test and more invasive than other tests because it sometimes requires sedation. Therefore, we consider that contrast-enhanced CT is the most useful test to diagnose thrombosis in the LSPV stump after LUL. Takeuchi et al. reported that 64-slice multidetector CT was useful to diagnose a floating thrombus in the PV [20],[21]. Contrast-enhanced CT is an easy and objective test, and it is also performed as follow-up after lung cancer resection. We recommend that contrast-enhanced CT be performed as early as possible at least once after LUL.

What is the treatment for thrombosis in the LSPV stump after LUL? Because it may easily enter the systemic circulation and cause arterial embolism, we recommend that anticoagulant therapy be started immediately. Additionally, based on the results of the present study, we also recommend anticoagulant therapy if intraoperative ultrasonography reveals SEC in the LSPV stump. If arterial embolism unfortunately develops, surgical removal of the thrombus should be considered. Ohira et al. reported that a case with thrombus in the LSPV stump developed cerebral infarction and underwent surgical removal of a thrombus [6].

Asai et al. reported that a case developed syncope after segmentectomy of the left upper division and showed a thrombus in the LSPV [22]. In this case, there was a thrombus in the main LSPV, not in the stump of the branches to the upper division. In this case, stricture of the LSPV secondary to stretch and deviation might be a cause of thrombus. There is a need for further discussion about thrombosis in the PV after segmentectomy.

The limitation of this study was that intraoperative ultrasonography was performed in the lateral decubitus position. To more closely approximate daily conditions, blood flow in the PV stump was evaluated after inflation of the affected lung. However, blood flow in the PV stump in the lateral decubitus position might not reflect that in the usual position for ultrasonography.

## Conclusions

By evaluating blood flow in the PV stumps using intraoperative ultrasonography, blood flow was found to be significantly slower in the LSPV stump than in the other three PVs. The patient who showed SEC in the LSPV stump intraoperatively developed thrombosis in the LSPV stump after LUL. In patients who undergo LUL, intraoperative ultrasonography to evaluate blood flow and the presence of SEC in the LSPV stump may be useful to predict thrombosis.

## Authors’ original submitted files for images

Below are the links to the authors’ original submitted files for images. Authors’ original file for figure 1 Authors’ original file for figure 2 Authors’ original file for figure 3 Authors’ original file for figure 4 Authors’ original file for figure 5

## Abbreviations

LUL: Left upper lobectomy
LSPV: Left superior pulmonary vein
PV: Pulmonary vein
PA: Pulmonary vein
RML: Right middle lobectomy
VATS: Video-assisted thoracoscopic surgery
POD: Postoperative day
SEC: Spontaneous echo contrast
LLL: Left lower lobectomy
RUL: Right upper lobectomy
RLL: Right lower lobectomy
LA: Left atrium
LAA: Left atrial appendage
