# Supplementary figures and images for: Blood stasis may cause thrombosis in the left superior pulmonary vein stump after left upper lobectomy
Source: J Cardiothorac Surg. 2014 Sep 18;9:159. doi: 10.1186/s13019-014-0159-8 (PMC4177051; doi:10.1186/s13019-014-0159-8)

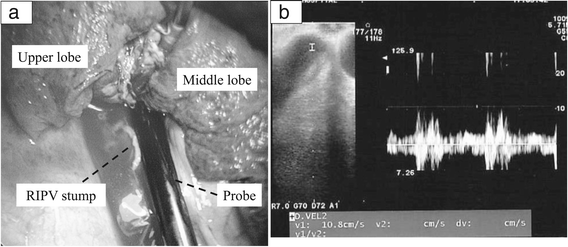

Supplement: Supplementary file 1 — Authors’ original file for figure 1 [file 13019_2014_159_MOESM1_ESM.gif]

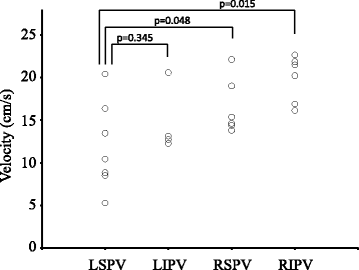

Supplement: Supplementary file 2 — Authors’ original file for figure 2 [file 13019_2014_159_MOESM2_ESM.gif]

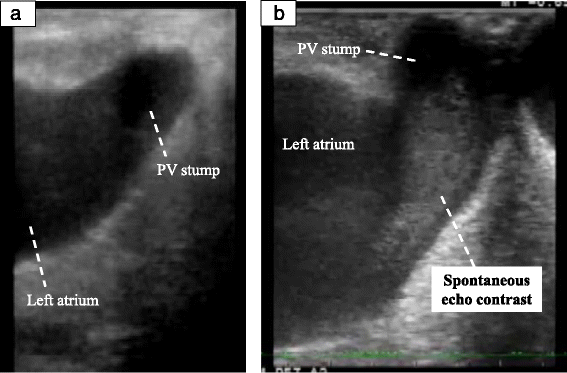

Supplement: Supplementary file 3 — Authors’ original file for figure 3 [file 13019_2014_159_MOESM3_ESM.gif]

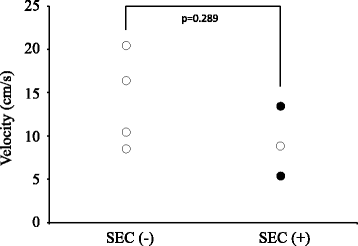

Supplement: Supplementary file 4 — Authors’ original file for figure 4 [file 13019_2014_159_MOESM4_ESM.gif]

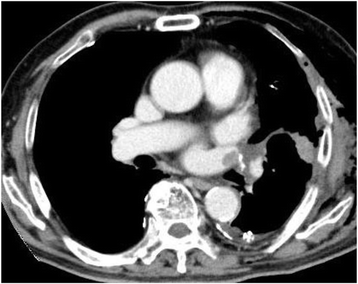

Supplement: Supplementary file 5 — Authors’ original file for figure 5 [file 13019_2014_159_MOESM5_ESM.gif]
